# The Role of Semantic Associations as a Metacognitive Cue in Creative Idea Generation

**DOI:** 10.3390/jintelligence11040059

**Published:** 2023-03-27

**Authors:** Yoed N. Kenett, Noam Gooz, Rakefet Ackerman

**Affiliations:** Faculty of Data and Decision Sciences, Technion—Israel Institute of Technology, Haifa 320003, Israel

**Keywords:** creativity, originality, metacognitive judgments, heuristic cues, semantic distance

## Abstract

Is my idea creative? This question directs investing in companies and choosing a research agenda. Following previous research, we focus on the originality of ideas and consider their association with self-assessments of idea generators regarding their own originality. We operationalize the originality score as the frequency (%) of each idea within a sample of participants and originality judgment as the self-assessment of this frequency. Initial evidence suggests that originality scores and originality judgments are produced by separate processes. As a result, originality judgments are prone to biases. So far, heuristic cues that lead to such biases are hardly known. We used methods from computational linguistics to examine the semantic distance as a potential heuristic cue underlying originality judgments. We examined the extent to which the semantic distance would contribute additional explanatory value in predicting originality scores and originality judgments, above and beyond cues known from previous research. In Experiment 1, we re-analyzed previous data that compared originality scores and originality judgments after adding the semantic distance of the generated ideas from the stimuli. We found that the semantic distance contributed to the gap between originality scores and originality judgments. In Experiment 2, we manipulated the examples given in task instructions to prime participants with two levels of idea originality and two levels of semantic distance. We replicated Experiment 1 in finding the semantic distance as a biasing factor for originality judgments. In addition, we found differences among the conditions in the extent of the bias. This study highlights the semantic distance as an unacknowledged metacognitive cue and demonstrates its biasing power for originality judgments.

## 1. Introduction

Metacognitive processes regulate our ongoing cognitive resources and processes. These metacognitive processes primarily consist of two levels. Object-level processes encompass basic cognitive operations, such as perceiving, remembering, classifying, and deciding; meta-level processes monitor the object-level operations (metacognitive monitoring) and allocate needed resources (metacognitive control; see Ackerman and Thompson 2017, for a review). Most metacognitive research has examined memory, reasoning, and decision making with well-defined tasks. Ill-defined tasks, such as creativity tasks, have been less studied under this framework (but see Puente-Díaz et al. 2021; Sidi et al. 2020). Thus, the exact role of metacognitive processes in creative thinking is largely unknown, especially heuristic cues that underly metacognitive judgments of originality.

A central question in metacognition is how people self-assess their confidence of success in each specific task item (e.g., an answer to a knowledge question). According to the well-established cue-utilization approach (Koriat 1997), people base their metacognitive judgments on various types of heuristic cues drawn from self-perception, information about the task and environment, and subjective experience while facing each task item (Ackerman 2019). Since metacognitive judgments rely on heuristic cues, they suffer from predictable biases stemming from differential effects of these cues on objective measures of success in the task and on the accompanied subjective self-judgments (Ackerman 2019; Bjork et al. 2013). As a result, the effectiveness of metacognitive control decisions which are based on biased judgments is expected to be compromised (Metcalfe and Finn 2008). 

In the current study, we aimed to expose biases in metacognitive judgments that accompany creative thinking. In particular, we were interested in the role of the conceptual distance between concepts as a cue for originality judgments. By that, we aimed to document the contribution of conceptual (i.e., semantic) distance to the gap between measured originality scores and originality judgments of participant responses in a creativity task.

### 1.1. The Role of the Semantic Distance in Creative Thinking

It is common to assess creativity with tests of divergent thinking (Acar and Runco 2019), by presenting ill-defined problems and asking people to generate creative solutions. The alternative uses task (AUT) is among the most common divergent thinking tasks used in creativity research (Acar and Runco 2019). In the AUT, participants are asked to come up with as many alternative uses as they can for common objects (e.g., bucket: light shade, stool; Guilford 1967). Several studies reported moderate to large correlations between AUT performance and real-world creative achievements in the arts and sciences (Jauk et al. 2014; Runco and Jaeger 2012).

Originality is among four typical components defining creative idea generation together with fluency, flexibility, and elaboration (Guilford 1967; Torrance 1966). The process of generating original ideas has been a focus of attention in creativity research (Runco and Jaeger 2012; Sowden et al. 2014). There is a range of approaches to assess the originality of ideas. Some of these approaches rely on experts’ evaluation (i.e., the Consensual Assessment Technique; Amabile 1983; Cseh and Jeffries 2019), some use standardized norms (Forthmann et al. 2020; Torrance 1972), and others involve subjective scoring by layperson judgments (Amabile 1983; Hass et al. 2018; Silvia et al. 2008). However, the need for tools allowing delving into biases in self-assessment of creativity is widely acknowledged (Kaufman 2019).

In recent years, creativity research has been increasingly moving towards the use of quantitative, objective assessment of AUT responses (Beaty and Johnson 2021; Dumas et al. 2021). These measures largely focus on the semantic distance. Based on the analysis of large textual corpora via computational linguistic models, semantic distance quantifies the conceptual dis-similarity between the AUT object, and the words in the open-ended responses participants generate when coming up with alternative uses for that object (Beaty and Johnson 2021; Dumas et al. 2021; Günther et al. 2019). Such measures correspond to the classic theory on memory, which argues that concepts in memory are organized as a semantic memory network, according to a principle of overlap of semantic features (Kumar 2021): The more semantic features two concepts share, the “closer” they are to each other in such a semantic memory network (Collins and Loftus 1975). Collins and Loftus (1975) defined the semantic distance as the ‘shortest path [direct or indirect] between two nodes’ (p. 412, note 3) within a person’s semantic memory network. They argue for a spreading activation model: once a concept in the semantic network is activated, activation spreads from it to all its directly connected neighbor concepts; an activation which quickly decays over time and space. 

The associative theory of creativity proposes that creative thinking is related to the ability to connect “remote” concepts in ones’ semantic memory network and that the activation of remote concepts results in ideas with higher novelty (Kenett and Faust 2019; Mednick 1962; Volle 2018). The associative theory of creativity has been supported recently by several studies of computational modeling. These studies documented higher connectivity of the semantic memory network structure among highly creative individuals relative to low creativity ones (e.g., Kenett and Faust 2019). In addition, multiple studies have found that such quantitative measures of the semantic distance strongly correlate with self-assessments of idea originality (Beaty and Johnson 2021; Forthmann et al. 2020). Overall, computing semantic distance scores from such large textual corpora is now extensively used in creativity research, as it has been shown to strongly capture individual differences in creativity (Kenett 2019, 2018). Yet, the role of executive processes, such as metacognitive processes, in guiding creative search and novelty generation is still far from understood (Beaty et al. 2014, 2022; Benedek et al. 2023; Ovando-Tellez et al. 2022a; Volle 2018).

### 1.2. Metacognitive Processes Underlying Creative Thinking

We aim to advance the understanding of creativity processes from a metacognitive point of view, in line with recent calls for cross fertilization between these tightly related domains (Lebuda and Benedek 2023; Puente-Díaz 2023) and neurological evidence associating the two (Rominger et al. 2022). Recently, Lebuda and Benedek (2023) proposed a comprehensive framework of creative metacognition. This framework consists of both dynamic and static metacognitive components, involving a core cognitive process utilizing knowledge, and two metacognitive processes focused on monitoring and control. In particular, the authors review literature demonstrating how people with higher creativity and intelligence are also more accurate in their originality judgments (Guo et al. 2022; Karwowski et al. 2020; Lebuda and Benedek 2023). 

In the current study, we used the AUT to expose biases when people judge their own originality. This is important because originality judgments are assumed to regulate creative efforts, similarly to other metacognitive judgments (Bjork et al. 2013; Fiedler et al. 2019; Perry-Smith and Mannucci 2017). That is, individuals are hypothesized to rely on originality judgments to guide their mental effort investment in further creative thinking (Puente-Díaz 2023). In this metacognitive causal chain, biased originality judgments, may lead people to terminate their idea generation process too early or keep investing labor in vain (Perry-Smith and Mannucci 2017). We focus on the semantic distance as a potential source for bias when people monitor their own originality. As explained above, semantic distance measures are based on large text corpora, quantifying relations between concepts that are largely shared across individuals (Günther et al. 2019; Mandera et al. 2015, 2017). This assumption underlies the large body of literature reviewed above, found to be robust and valuable in quantitatively assessing the originality of responses generated by participants in creativity tasks (Beaty and Johnson 2021; Dumas et al. 2021; Kenett 2019). We adapt this notion to the metacognitive realm. 

In metacognitive research, shared semantic structure across people has been broadly used to identify the heuristic cues that underly and may bias metacognitive judgments (Ackerman 2019). In particular, Koriat (1993) defined accessibility as the amount of associations that come to mind when encountering a knowledge question. For instance, Koriat and Levy-Sadot (2001) compared people’s metacognitive judgments regarding knowledge of composers (people generally know quite many names) versus choreographs (people generally know only a few). They found higher metacognitive judgments regardless of knowledge of a particular fact regarding a question when referring to a large body of knowledge (composers) relative to a small body of knowledge (choreographs). They also dissociated this number of associations from familiarity of the same terms. Ackerman and Beller (2017) applied this principle to problem solving and exposed similar biases in peoples’ responses in the compound remote associates task, a commonly used task to study convergent thinking in creativity research (Bowden and Jung-Beeman 2003; Wu et al. 2020).

### 1.3. The Present Study 

The present study is based on Sidi et al. (2020), who were the first to quantify the creative process in AUT in a manner that allowed examining metacognitive processes that underly originality. They operationalized originality as the percentage of participants in the study sample who suggested a similar idea to an object in the AUT. It is well established in the creativity literature that when people produce a list of potential ideas anchored to a single object, they start their ideation process with more mundane ideas, and get to the more original ideas later in the ideation process (Bai et al. 2021; Beaty and Silvia 2012). Sidi et al. (2020) replicated this finding. They collected originality judgments for each idea by asking participants to assess the percentage of participants that they expect will come up with the same idea as their own, in parallel to the definition of the originality score in their study. They found that originality judgments reflected the serial order properly, that is, people assessed their initial ideas as less original than their later AUT responses. Thus, the serial order was a reliable cue for originality judgments. Notably, the authors dissociated effects on originality scores and originality judgments. In their study, Sidi et al. (2020) manipulated originality scores without affecting originality judgments by providing information regarding the expected number of uses (low—2 or high—6); making the participants generate more or fewer ideas without acknowledging the changes in originality that it created. Demonstrating the other side of a double dissociation, they manipulated originality judgments but not originality scores by providing positive or negative feedback during the initial task practice. Thus, this study shows separated underlying processes for originality performance and its self-assessment. Particularly relevant for the present study is that this study demonstrates the sensitivity of both idea originality and originality judgments to instruction manipulations. 

Our aim was to examine the influence of semantic distance on the discrepancy between self-assessment of originality and the originality of ideas. We hypothesized that semantic distance is a heuristic cue underlying originality judgment, but that its role in originality judgments may not correspond to its role as a predictive cue of the rarity of an idea (originality score) in a target population. This hypothesis was examined in Experiment 1, by re-analyzing Sidi et al. (2020) data while adding a semantic distance variable estimated using computational semantics tools. In Experiment 2, we manipulated task instructions as priming for systematically affecting the generated ideas. In particular, the instructions introduced examples representing a combination of the semantic distance (close/far) and originality (low/high). This 2 × 2 design allowed us to examine the unique contributions of the semantic distance separately from the effects of originality scores when predicting originality judgments. Any interaction effect between the two manipulations on cues that underlie the discrepancy between self-assessment of originality and the originality of ideas would indicate their separability. That is, although semantic distance is strongly associated with originality, they are not identical, and it may have differential effects on objective and subjective aspects of the creative process. 

## 2. Experiment 1

### 2.1. Introduction

In Experiment 1, we examined the contribution of semantic distance to the explained variance of originality scores and originality judgments above and beyond other known influencing factors. We re-analyzed data from Sidi et al. (2020) Experiment 2 and Experiment 3. We refer to these experiments as 1a and 1b, respectively. In these experiments, participants performed the AUT with five objects after instructions and demonstrations with one object. For each object, participants could generate as many ideas as they wished. Immediately after generating each idea, they rated the originality of that idea on a 0–100% scale, representing the assessed rate of peers in the sample who generated the same idea. The dependent variables in both experiments were originality scores (the frequency of each idea in the sample) and originality judgments. Sidi et al. (2020) dissociated originality judgments and originality scores of responses with two manipulations, one that affected only the originality scores of the ideas (information about the number of ideas previous pseudo participants provided on average to each object being 2 or 6) and another one that affected only the judgments (positive/negative feedback).

We considered four heuristic cues that could potentially predict originality scores and originality judgments as independent variables. We examined response times (RT) and the serial order from Sidi et al. (2020) data, and added the number of words, as a proxy to idea specificity, and the semantic distance derived from the SemDis platform, for every generated idea. We hypothesized that the semantic distance would predict both originality scores and originality judgments. However, we expected to find a strong association between the semantic distance and originality scores based on the creativity literature that uses semantic distance measures as a direct measure of originality (Beaty and Johnson 2021; Dumas et al. 2021; Kenett 2019). Given that responses’ SemDis scores are derived from the analysis of aggregated, large-volume text-corpora, we expected judgments of originality to be affected more by other cues and less by the semantic distance relative to predicting originality scores. This differential strength of effects for originality scores and originality judgments was expected to explain part of the discrepancy between the two, and thus expose the semantic distance as a factor impacting behavior in the AUT.

For the main data analyses of cue contribution and biasing role in originality scores and originality judgments, we used a recently suggested methodology called Bird’s-Eye View of Cue Integration (BEVoCI; Ackerman, forthcoming current version is available in the OSF page of this paper). It allows to dissociate factors affecting achievements (cue validity, Koriat 1997) and judgments (cue utilization, Koriat 1997). A cue is considered misleading when it predicts achievements and judgments differently (also considered as a bias). Such a difference in prediction can be expressed by a positive, negative, or no effect on one dependent variable (e.g., performance or judgments) but not on the other (Ackerman forthcoming). Furthermore, another possibility for such cue misleading effect is related to differential predictive power (as represented by regression β) in one dependent variable compared to the other. Notably, most metacognitive experiments which demonstrate double dissociations between judgments and performance manipulated one biasing factor a time, in one experiment with one affecting judgment and not performance, and in the other affecting performance and not judgments (e.g., Metcalfe and Finn 2008; Sidi et al. 2020). The BEVoCI, in contrast, allows examining several potentially misleading cues and exposing double dissociations in the same analysis with the same sample.

The BEVoCI method is based on two hierarchical multiple regressions, one predicts an objective measure of performance by multiple considered cues, and the other one predicts a metacognitive judgment by the same cues. Then, the two sets of predictive values are compared in direction and strength of the effect for each cue. The hierarchical nature of the analyses allows to statistically compare groups of participants while taking into account the unique cue weights for each participant, and the weights’ variance among the participants in each condition. 

In this study, we applied the BEVoCI method for exposing the unique contribution of the semantic distance, word count as a control variable, as well as additional cues examined in metacognitive research (RT and serial order) in predicting originality scores and originality judgments. In particular, the BEVoCI method allows exposing the differential effects of each cue on the two dependent variables. Recent studies suggest that people integrate four and even five cues in their memory judgments and in confidence in problem solutions (Ackerman forthcoming; Undorf et al. 2018; Undorf and Bröder 2021). The BEVoCI method allows examining whether such multiple cue integration is reflected in originality judgments as well.

### 2.2. Materials and Methods

#### 2.2.1. Participants

Sidi et al. (2020) experiments were conducted in English with an international online sample collected from Prolific.ac (71% females, Mage = 30.7 years, SD = 5.4 years, *N* = 101 and *N* = 96 participants in the two experiments, respectively) in exchange for GBP 1.3 for each participant.

#### 2.2.2. Materials

##### The Alternative Uses Task (AUT)

The AUT involves presenting an object and asking for a sequence of potential uses for it (Acar and Runco 2019; Runco and Acar 2012). Sidi et al. (2020) used brick as an example. The main task’s objects were: bucket, cloth hanger, wine cork, paperclip, and shoelace. Each object appeared with its name and a picture. Each trial was self-paced, and participants could generate as many AUT responses as they wanted, before moving on to the next trial.

##### Measures

**Originality scores** for each AUT response were calculated as percentages based on the following process: Two trained raters coded all of the participants’ ideas according to a predefined coding scheme. Inter-reliability between the raters was strong (Cohen’s K = 0.867, *p* < 0.001). The frequency (%) of each idea within the experiment’s sample for each object (e.g., brick) was calculated. In this way, the higher the score, the more unoriginal (more common) the generated response was. The originality (uniqueness) score can thus be measured by taking the inverse of the frequency scores (100%- frequency).

**Originality judgments** were elicited for each idea on a scale of 0–100%. The higher the judgment, the more unoriginal (more common) the participant judged their response to be. Therefore, we inversed these ratings to indicate originality by calculating 100-unoriginality judgment.

**The response time** (RT) and the **serial order** of responses were documented by the experiment software. RT measures the time elapsed from page presentation until participants entered their ideas. The serial order reflects the location within the idea stream of each participant for each object. 

**The number of words** in each response was counted, as it is a known confounding factor for originality in the creativity literature (Forthmann et al. 2019).

**The semantic distance** of AUT responses was calculated through the SemDis platform (Beaty and Johnson 2021; http://semdis.wlu.psu.edu/, accessed on 1 May 2022), which is a publicly available application for calculating the semantic distance of AUT responses. This tool operationalizes the theoretical concept of the semantic distance to a measurable dependent variable. We used the additive calculation procedure to compute the semantic distance between each participant’s response relative to the presented object (e.g., Brick). This calculation is performed based on an average score over five different semantic spaces, mitigating the effects of a single semantic model and text corpus (e.g., textbooks vs. movie subtitles; Beaty and Johnson 2021; Dumas et al. 2021; Kenett 2019). Three of these spaces are built upon continuous bag of words (CBOW) prediction models (cbowukwacsubtitle, cbowsubtitle, and cbowBNCwikiukwac) and two are built upon count models (GloVe and TASA). The CBOW models use a neural network architecture (Mandera et al. 2017) that predicts a given word from surrounding context words within a given text corpus (for more details see Beaty and Johnson 2021). 

##### Analyses

**Bird’s-Eye View on Cue Integration** (BEVoCI). The analyses start by examining co-linearity among the considered cues. Only cues with sufficient unique variance are included in the models, for exposing their unique contribution while controlling for all other cues. As a rule of thumb, Ackerman (forthcoming) suggested using *r* < 0.30 in all paired correlations as a threshold for including cues. 

Two hierarchical multiple regression models were used with level 1: objects; level 2: participants. The analyses were performed by R 4.1.0 lmer package (Pinheiro et al. 2019). Prior to the analyses, we verified that all participants had variability in originality judgments and originality scores. Then, all independent and dependent variables were standardized. All cues were entered into one model predicting originality scores and another one predicting originality judgments. In these models, we controlled for participant and object variability. 

#### 2.2.3. Procedure

In both re-analyzed experiments, participants completed the AUT in two conditions to which participants were randomly assigned. The instructions used a picture of a brick to demonstrate the task and the user interface for entering each use idea. The idea elicitation phrasing was: “Please come up with as many uses as you can think of for a brick.” Accompanied by a picture of a brick. Assessing originality was elicited with the following: “Please assess how many participants (%) came up with this use” with a continuous scale, with captions of “Nobody else” near the 0%, and “Everybody” near the 100%. Participants could produce as many ideas as they wished. Notably, a request to be original was not mentioned. 

### 2.3. Results

First, for each re-analyzed experiment, we correlated all the predictors and found that in Experiment 1b RT had multicollinearity with the number of words (*r* > 0.3). We chose to exclude RT and keep the number of words for controlling for this potential confounder (Forthmann et al. 2019) and obtaining cleaner analyses of the semantic distance. Next, we applied the BEVoCI method on both re-analyzed experiments from Sidi et al. (2020) with serial order, number of words, and semantic distance as cues for both experiments, and RT for Experiment 1a only. See Table 1 the beta coefficients of the two regression analyses performed for each group in Experiment 1a and Experiment 1b.

Including semantic distance in BEVoCI analyses revealed that semantic distance, and no other cue, generated a significant difference in predicting originality scores and originality judgments in three out of four conditions (Table 1), while in the fourth condition the bias was trending towards significant, *t*(1696) = 1.76, *p* = 0.078. Comparing the two dependent variables when including the entire data set of Experiment 1a, without division into conditions, revealed a significant difference in the role of the semantic distance between them, *t*(3404) = 3.09, *p* = 0.002. The stronger predictive power for originality scores (higher β) than for originality judgments (Table 1) suggests that the semantic distance was underutilized for originality judgments. The found difference might either stem from different “slopes” when predicting each dependent variable—originality scores and originality judgments—by semantic distance while controlling for other cues, from the amount of “noise” around each prediction, or a combination of the two. Standard errors of the βs were 0.029 for both dependent variables, ruling out the “noise” difference as an explanation for the mismatch between the two. 

### 2.4. Discussion

In Experiment 1, we re-analyzed data from Sidi et al. (2020). In addition to the robust serial order effect, they reported on, we considered the potential utilization of semantic distance as a predictor of originality scores and as a cue for originality judgments, while controlling for the number of words and response time. All considered cues had predictive power in all models. This finding should certainly not be taken for granted. Importantly, in most models, semantic distance was underutilized in originality judgments, unlike all other considered cues. In Experiment 2, we delved experimentally into the effects of the semantic distance on these aspects of creativity.

## 3. Experiment 2

### 3.1. Introduction

In Experiment 2, we aimed to delve into the role of semantic distance in metacognitive processes underlying originality behavior. In creativity research, semantic distance is increasingly used as a quantitative, objective measure assessing originality (e.g., Beaty and Johnson 2021; Dumas et al. 2021; Kenett 2019). The results of Experiment 1 revealed that semantic distance was a strong predictor of originality scores, and to a lesser extent of originality judgments. These findings thus lead to the hypothesis that semantic distance captures differently originality scores than originality judgments. To examine this hypothesis, we manipulated the instructions for the AUT to dissociate the priming of semantic distance from priming for originality, in a 2 × 2 between-participants design.

As part of the instructions, we provided three examples of objects, different than those appearing in the main task. For each object, we gave one example of a use for this object. Based on responses given by participants in Sidi et al. (2020) study, we chose examples of uses by two orthogonal factors, the semantic distance (close or far) and originality (low or high; see Table 2). All three examples in each condition had the same combination of the semantic distance and originality. Notably, we included the seemingly contradictory conditions of a high semantic distance with low originality and vice versa, to highlight the separability between the two.

Our focus is on the predictive value of the examined cues. Overall, given the positive direction of the prediction by semantic distance in Experiment 1, we hypothesized that originality scores and originality judgments would rise as semantic distance increases. Moreover, we expected originality scores to be more sensitive to semantic distance manipulation than originality judgments. This is because semantic distances are calculated based on a generalized model from large textual corpora, originality scores are calculated based on co-occurrence statistics of words in the corpora, and originality judgments are idiosyncratic in nature. This explanation was indeed supported by the findings of Experiment 1 and was expected to be replicated in Experiment 2.

As for the manipulations, we expected two types of effects. The first was shifts in means of originality scores and originality judgments with both manipulations. The second was shifts in “slopes” (β values), in particular those related to semantic distance. Using BEVoCI analyses was expected to expose changes in cue validity and cue utilization given the manipulations we used. As explained above, any interaction effect of the two manipulations would indicate the unique contributions of the two manipulations to the creative process.

### 3.2. Materials and Methods

#### 3.2.1. Participants

In Experiment 1, Sidi et al. (2020) had approximately 50 participants in each group, in one group, the differential effect of the semantic distance on the originality score and originality judgment was marginal, while in others it was significant. Thus, we increased the power by approximately 20–25%, in order to increase the robustness of Experiment 2. The experiment was conducted with an international online sample from Prolific.ac. In total, 262 participants took part in exchange for 2.5 GBP. They were invited for approximately 30 min. They were assigned randomly to one of four study groups (2 × 2 between subject manipulation). In each group, we had 61–65 participants after 10 participants were excluded from the data following failure to answer two or more of the following attention checks: (1) instruction understanding verification by a multiple choice question, inferring a detail from a displayed example; (2) clicking “I do not remember the use I entered” when rating originality on the following screen more than once; (3) or failing upon instruction to input a particular value to two statements in a final section of self-report questions. After exclusion, 252 participants (76% females, mean age = 34 years, *SD* = 11.6 years) were included in the analyses. 

#### 3.2.2. Materials

##### Alternative Uses Task (AUT)

Similarly to Experiment 1, we used the AUT. Participants were asked to generate alternative uses for nine objects: hammer, lipstick, fork, bench, apron, tie, pillow, knife, and belt. The chosen objects for the AUT reflected the nine psycholinguistic dimensions scales of the Glasgow Norms (arousal, valence, dominance, concreteness, imageability, familiarity, age of acquisition, semantic size, and gender association) to avoid linguistic biases (Scott et al. 2019). The objects were used in the same manner as in Sidi et al. (2020). In addition, we prepared three example objects with four uses each (Table 2). The uses for the example objects represented the four conditions: original or common uses which were either far or close in terms of semantic distances. The semantic distances of the examples were computed by the “fasttext” model in Python (Joulin et al. 2016) relative to the respective object word (e.g., bucket). The examples with high or low originality scores were taken from responses provided by the participants in Sidi et al. (2020; Experiment 2). Original examples were chosen from the first quartile of originality scores and common examples were chosen from the last quartile for each example object. For example, a closely semantic and original idea for a bucket was a shovel, which was one step away from bucket in the semantic network and was generated by only 1% of the participants of Sidi et al. (2020).

Notably, we deliberately phrased the use examples to be shorter in the number of words (*M* = 2.77 words, *SD* = 1.24) than the responses provided by Sidi et al.’s (2020) participants (*M* = 3.44 words, *SD* = 2.52). By that, we aimed to focus participants on their central use idea, to improve word count and semantic distance reliability relative to the previous study by minimizing peripheral words (e.g., “the” or ”a”). We also expected that a shorter description of uses would encourage participants to provide more uses for each object without investing a longer time. This was expected to increase our study power and increase the explanatory value of the serial order.

##### Measures

The independent and dependent variables were the same as those used in Experiment 1. Unlike in Sidi et al. (2020), given the brief idea phrasings, originality scores were calculated by the frequency in the overall sample of each idea’s dominant word, which was manually chosen by one of the authors (NG). The considered predictive cues were as in Experiment 1: serial order, number of words in the use idea, semantic distance of the use from the object, and RT.

##### Analyses

We started with examining the effects of the two manipulations on means of originality scores and originality judgments by two-way analyses of variance (ANOVA), with the semantic distance (close vs. far) and originality (low vs. high) as between-participant factors. Then, we performed the BEVoCI analyses for each condition separately, as in Experiment 1. Finally, unlike in Experiment 1, we compared cue validity and cue utilization (β coefficients) among the groups by adding the two manipulations and the interaction between them as additional factors in BEVoCI regression models.

#### 3.2.3. Procedure

**Figure 1:**
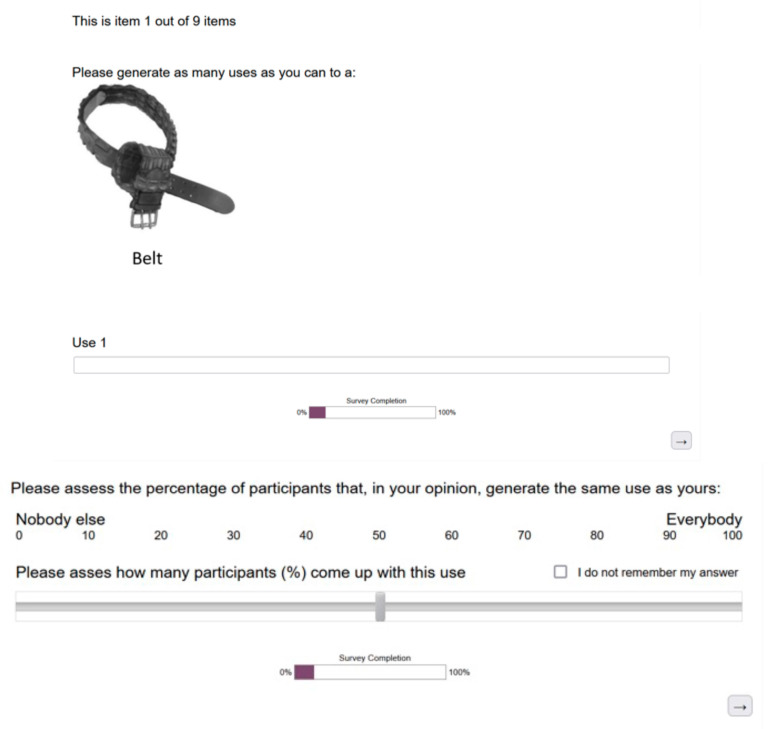
An example of a trial in the AUT used in Experiment 2.

We used the AUT with nine objects accompanied by images, presented as in Experiment 1. Participants were required to generate at least one use idea, before moving to the next object. The task was self-paced and participants could generate as many use ideas as they wanted for each object. See a screenshot of one trial in Figure 1.

#### 3.2.4. Results

We start by examining the effects of the manipulations on the means of the dependent variables. Comparing the means of the dependent variables across the four conditions revealed that our semantic distance manipulation did not affect the means of originality scores, *F*(1, 248) = 0.44, MSE = 18.1, *p* = 0.51, η^2^_p_ = 0.002, nor originality judgments, *F*(1, 248) = 1.28, MSE = 293.3, *p* = 0.26, η^2^_p_ = 0.005. When the prime was close in terms of the semantic distance, uses were generated with slower RT (*M* = 12.4 sec., *SD* = 7.0) than when it was semantically far from the given object (*M* = 10.9 sec., *SD* = 5.4), *F*(1, 248) = 3.95, MSE = 154.4, *p* = 0.048, η^2^_p_ = 0.016. Our manipulation of originality affected originality scores, *F*(1, 248) = 15.76, MSE = 640.7, *p* < 0.001, η^2^_p_ = 0.060, with groups primed with more original examples (*M* = 82.0, *SD* = 5.7) generating more original ideas than the those who received less original examples (*M* = 78.6, *SD* = 5.8). No parallel effect of originality manipulation on means of originality judgments was found, *F*(1, 248) = 0.58, MSE = 133.5, *p* = 0.45, η^2^_p_ = 0.002, or on RT, *F*(1, 248) = 0.01, MSE = 0.35, *p* = 0.925, η^2^_p_ = 0.000. Finally, no interactive effects between the semantic distance and originality were found for originality scores, *F*(1, 248) = 0.64, MSE = 2.6, *p* = 0.80, η^2^_p_ = 0.000, originality judgments, *F*(1, 248) = 0.62, MSE = 141.3, *p* = 0.43, η^2^_p_ = 0.002, or RT, *F*(1, 248) = 0.13, MSE = 5.05, *p* = 0.720, η^2^_p_ = 0.001. Notably, the effect of the originality manipulation on originality scores but not on originality judgments already exposes a dissociation between them.

Next, we applied the BEVoCI analysis for examining cue validity and cue utilization. There was no collinearity of above 0.30 correlations among the considered cues. The BEVoCI results for Experiment 2 are presented in Table 1. All cues were significant predictors for both originality scores and originality judgments in most models. RT was unique in being a non-significant predictor in some cases. Given that it has no unique contribution in Experiment 1b due to collinearity, we conclude that it is a secondary, if at all, contributor to originality behavior above and beyond the other cues we considered.

**Figure 2:**
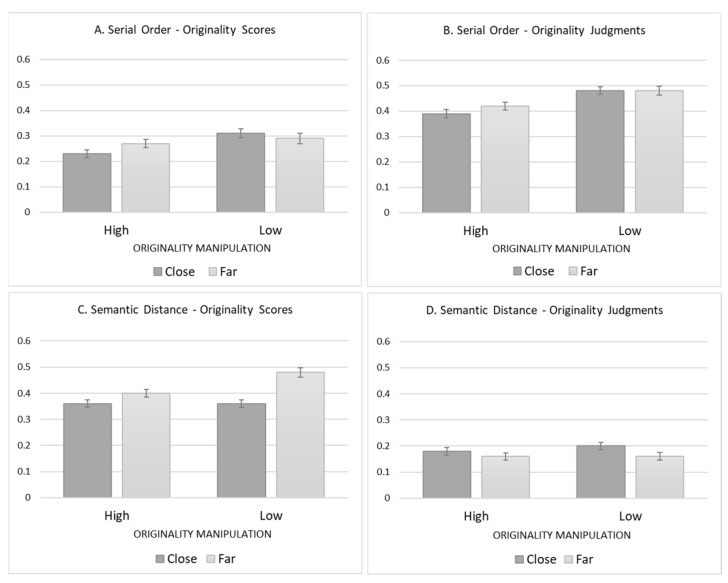
β of originality scores and originality judgments in the four experimental groups by the two cues that generated biases, the serial order, and the semantic distance. Error bars represent the standard error of the means for the β’s.

Consistent with Experiment 1, in all BEVoCI models, semantic distance contributed to the mismatch between originality scores and originality judgments, as it was a stronger predictor of originality scores than originality judgments. Interestingly, and unlike Experiment 1, in all BEVoCI models, serial order contributed to this mismatch as well, with the opposite effect: a weaker predictive power for originality scores than for originality judgments. Notably, the β of the serial order for originality judgments was generally higher in Experiment 2 relative to Experiment 1. We explain this finding in the shorter responses participants provided when primed with brief examples and the production of more responses in Experiment 2 relative to Experiment 1. However, since no parallel difference in values was found regarding originality scores, it seems that the serial order affected originality judgment more than ideas’ originality. Indeed, while semantic distance was consistently underutilized, serial order was consistently overutilized (see graphical representation by the β bars in Figure 2). These opposite directions support the conclusion of Sidi et al. (2020) that originality scores and originality judgment are dissociated as they are affected by different underlying processes.

Next, we analyzed the effects of the 2 × 2 manipulations on β for each cue separately. This was performed to examine the effect of the manipulations on the predictive power of each of the cues on originality scores and originality judgments. These regression analyses revealed several main effects and interaction effects (Table 3). As explained in our hypotheses above, the main finding is the mere existence of interactions between the two manipulations. These interactions indicate the separability and unique contribution of semantic distance manipulation on top of the effects of the originality manipulation. This finding goes beyond the effects on the means of originality scores. Interestingly, we see an effect of the originality manipulation: a higher overall serial order β (regardless of distance manipulation) of originality judgments; a higher originality score β in the far semantic distance manipulation than all others.

### 3.3. Discussion

In Experiment 2, we replicated the main findings from Experiment 1 regarding semantic distance. We found that semantic distance was a strong predictor of both originality scores and originality judgments and was a cue that consistently predicted originality scores more strongly than originality judgments. In addition, we exposed serial order effects that were not found in Experiment 1. Interestingly, this effect was the opposite of the effect of semantic distance, as serial order was consistently a weaker predictor of originality scores than of originality judgments. Moreover, our priming manipulation demonstrated the dissociation between originality scores and originality judgments, as manipulating the originality of the examples within the instructions only affected originality scores and not originality judgments. Unlike previous studies (Heinen and Johnson 2018), our semantic distance manipulation of the examples in the AUT instructions did not lead to any significant differences in originality scores or originality judgments. However, this manipulation did affect the predictive value of the semantic distance and the serial order.

## 4. General Discussion

In the current study, we examined the role of semantic distance in self-evaluation of creative ideas. To do so, we re-analyzed data collected by Sidi et al. (2020) and conducted a follow-up experiment. Sidi et al. (2020) manipulated originality scores separately from originality judgments (by manipulating the expected number of generated ideas) and vice versa (by manipulating the feedback during instructions). Their findings support double dissociation between effects on performance and judgments by using a separate manipulation for each side of the dissociation. However, by considering more cues and using the BEVoCI analysis approach, our study highlights key insights for both metacognitive and creativity research.

In both experiments, we considered serial order, based on Sidi et al. (2020), and added cues from creativity literature, namely, number of words and semantic distance. We also examined RT which is often used in metacognitive research (e.g., Ackerman and Zalmanov 2012), in order to examine the unique contribution of semantic distance above and beyond well-established cues. All considered cues were found to be significant predictors of both originality scores and originality judgments in most cases. Examining the unique contribution of semantic distance led us to discover that it was a strong predictor for both originality scores and originality judgments. However, it also had a consistent biasing effect, which is a novel finding in both metacognitive and creativity research.

First, across both experiments, we found that semantic distance of AUT responses, operationalized via SemDis, is a significant predictor of originality judgments, and replicated prior work demonstrating its ability to predict originality scores (Beaty and Johnson 2021; Dumas et al. 2021; Kenett 2019). We emphasize the dissociative effect of semantic distance on originality scores versus originality judgments, highlighting a bias in the utilization of this cue. In Experiment 1 of the present study, semantic distance affected originality scores more than originality judgments. In Experiment 2, we replicated this finding and also found that serial order affected originality scores less strongly than originality judgments. These findings suggest that originality judgments, as with many other metacognitive judgments (e.g., judgment of learning, confidence; Ackerman 2019), are based on heuristic cues. Furthermore, these findings suggest that originality judgments’ correspondence with behavioral performance depends heavily on the validity of the cues people utilize for inferring them (Ackerman 2019; Koriat 1997). By manipulating semantic distance and the originality of examples as two different factors in Experiment 2, our findings uniquely highlight that semantic distance and originality scores do not fully correspond to each other, although they are consistently correlated.

Second, in line with previous research with other metacognitive judgments (Ackerman forthcoming; Undorf et al. 2018; Undorf and Bröder 2021), we found cue integration of four cues in originality judgments. The fact that BEVoCI allows to directly compare the predictive role of a quantitative measure of the semantic distance relative to other cues advances metacognitive and creativity research both theoretically and methodologically (Beaty and Johnson 2021; Dumas et al. 2021; Kenett 2019). This relative comparison revealed that semantic distance was the strongest among the cues we considered to predict originality scores. However, in regard to predicting originality judgments, semantic distance was as strong as serial order in Experiment 1, and a weaker predictor compared to serial order in Experiment 2. A future direction of interest is to examine whether this trade-off between the two cues can be replicated and generalized.

Third, most metacognitive research regarding cue utilization deals with indicators of item difficulty that are accessible to the participant while performing the task (e.g., cue familiarity, text coherence; Ackerman 2019). In the present study, serial order and RT are cues of this type. In contrast, responses’ SemDis scores are derived from the analysis of non-idiosyncratic, aggregated, large-volumes text-corpora (Günther et al. 2019). Indeed, as expected, originality judgments were less affected by semantic distance compared to originality scores. This is a unique situation in metacognitive research that contributes a new direction for considering other such information sources. Notably, though, although the semantic distance is defined based on information external to the participant, it is assumed to reflect knowledge structure common across people with similar cultural backgrounds (Günther et al. 2019; Mandera et al. 2015, 2017). Even more radical in this continuum of personalization is consensuality (Koriat 2008). Consensuality is the extent to which the same response is given within the experiment sample. It is not expected to be accessible to each participant but was found empirically to predict metacognitive judgments in various contexts (Ackerman et al. 2019, 2020; Bajšanski and Žauhar 2019). Consensuality is highly similar to the way we operationalized originality scores. In the present study, we highlight differences between the consensus, measured by originality scores, and the related metacognitive judgments, as reflected by originality judgments.

Finally, in Experiment 2, we conducted an instruction manipulation, manipulating different aspects of originality (low, high) and semantic distance (close, far) of the examples provided for the AUT and examined the effects of these manipulations on participants’ performance. Despite previous studies demonstrating manipulations of the semantic distance of responses based on instruction manipulation (Heinen and Johnson 2018), we did not find significant effects on originality scores and originality judgment means. This finding may indicate power issues of our manipulation. However, these two manipulations (originality and semantic distance) affected participants’ sensitivity to the considered metacognitive cues. This is evident in significant changes in regression slopes, as estimated via the models’ β as reported in Table 3. Using state-of-the-art statistical methods, such as BEVoCi, allows unpacking more nuanced effects of such manipulations on performance and judgments in complex tasks such as the AUT.

Ill-defined tasks, such as creativity tasks, have been rarely studied under the metacognitive framework (but see Puente-Díaz et al. 2021; Sidi et al. 2020). Thus, the exact role of metacognitive processes in creative thinking is largely unknown, especially the heuristic cues underlying metacognitive judgments of originality (Lebuda and Benedek 2023). Our results highlight the complexity of the cognitive and metacognitive capacities involved in generating creative responses. More broadly, we found that each manipulation affected more strongly different cues. We suspect that this is only the tip of the iceberg and that there are more cues involved in creative processes still to be exposed. For example, Matheson and Kenett (2021) demonstrated how different motion-related strategies impact AUT responses (see also Gilhooly et al. 2007). In a recent study of theirs, Sargent et al. (2023) empirically demonstrated how environmental context and body posture impact performance on the AUT. It is yet unknown how such novel aspects affect metacognitive judgments that accompany the thinking process.

We would like to point to several methodological aspects of our study regarding how we administered the AUT. First, the way the AUT was administered in both experiments varies from common implementation in creativity research (Said-Metwaly et al. 2020). Most creativity studies administer the AUT with time constraints (Paek et al. 2021), such as two or three minutes per object, whereas we applied a self-paced AUT. Furthermore, in both experiments, originality scores were computed as the inverse of the commonality of responses in the sample, based on the approach of Sidi et al. (2020). It is possible, that asking about commonality and about rarity differ in their underlying cues, while the objective measure of originality remains unchanged. Moreover, originality scores are typically assessed subjectively, by having raters rate the originality of responses (Hass et al. 2018; Silvia et al. 2008). These judgments may share different aspects with self-judgments than with the objective operationalization of originality scores that we used, as those subjective judgments of other people, are probably heuristic based as well. Notably, such frequency-based approaches are known to suffer from reliability limitations (Forthmann et al. 2021; Silvia et al. 2008), further highlighting the need to improve assessment of idea originality (Kaufman 2019). In addition, typical creativity research using the AUT instructs participants to “be creative” in their responses (Nusbaum et al. 2014), while we refrained from any such instructions so as to not bias metacognitive processes. Finally, the instructions given in Experiment 2 used short responses as examples and this change relative to Experiment 1 seems to affect ideation fluency. This design aspect potentially limited the richness of the AUT responses, impacting their elaboration that we measured via the number of words. All these methodological aspects worth consideration in future research.

A core aspect of our current study is the computation of semantic distance scores between objects and the alternative uses generated to these objects by participants. This computation was performed via the SemDis platform (Beaty and Johnson 2021), which is based on natural language processing models that derive similarity between concepts based on co-occurrence statistics in large textual-corpora (Günther et al. 2019; Kumar 2021; Mandera et al. 2015). Semantic distance—the inverse of similarity between concepts in such a semantic space—has been consistently shown to be a quantitative, objective measure of idea originality (Beaty and Johnson 2021; Dumas et al. 2021) compared to traditional, subjective scoring (Silvia et al. 2008). Such aggregated text-based measures of the semantic distance are extremely useful in capturing commonalities across people and individual differences among them (Beaty and Johnson 2021; Dumas et al. 2021). Critically, such methods have been extremely useful in studying the role of semantic memory in creative thinking (Abraham and Bubic 2015; Benedek et al. 2023). As mentioned above, assumptions regarding common knowledge structure across people are well-established in metacognitive research as well (Ackerman and Beller 2017; Koriat 1993; Koriat and Levy-Sadot 2001). However, two potential limitations of such text-based methods are that they are based on aggregated large textual corpora that may not fully map onto each participants’ semantic knowledge structure (Kenett 2019). Thus, future research should replicate and extend our current findings by applying methods to estimate individual-based semantic memory networks (Benedek et al. 2017; He et al. 2020; Ovando-Tellez et al. 2022b; Wulff et al. 2022). Such a design would allow matching individual-based originality scores (based on the semantic distance in their own semantic memory network) and originality judgments.

More generally, our findings highlight the role of metacognitive processes in creative thinking, which are still largely unknown (Puente-Díaz 2023). Our results replicate previous findings (Sidi et al. 2020) that people consistently underestimate their originality, while also judging reliably their initial ideas as more creative than their later ones (serial order effect). However, our Experiment 2 demonstrated that people sometimes rely on the serial order too strongly, on the semantic distance too little, and their creative process is affected by examples that vary in originality and the semantic distance. This collection of findings conveys insights for real-life applications. For example, encouraging higher originality judgments may better align them with the remoteness of the later responses and could also facilitate generation of more creative ideas. Furthermore, Lloyd-Cox et al. (Lloyd-Cox et al. 2022) recently showed how when people evaluate the creativity of AUT responses they over-emphasize novelty, while when evaluating the creativity of solutions to real-life problems they over-emphasize appropriateness. Similarly to Lloyd-Cox et al. (Lloyd-Cox et al. 2022), our findings demonstrate malleability of cue validity (predicting objective performance) and cue utilization (predicting judgments; Koriat 1997; Runco and Acar 2012) in creative tasks, contingent on various task demands and contexts. Finally, while we did not instruct people to “be creative” in their responses, such instructions are quite impactful on people’s responses in the AUT, especially compared to “be-fluent” instructions (Forthmann et al. 2016; Niu and Liu 2009). Given the dissociation we found between originality scores and originality judgments, future studies of creativity may also consider examining a metacognitive emphasis (e.g., “be-confident” type) as part of the instructions.

## 5. Conclusions

The present study takes an empirical step forward in converging creativity and metacognitive research. So far, little is known about the role of metacognitive processes in creative thinking (Lebuda and Benedek 2023), and the potential dissociation between performance and judgments in creativity tasks (Benedek et al. 2016; Sidi et al. 2020). Our study uniquely shows the role of semantic distance as a metacognitive cue affecting originality judgments, its role as a biasing metacognitive cue in creativity, and a double dissociation between the opposite utilization effects of semantic distance and serial order on originality scores and originality judgments. Overall, our work highlights the need for further empirical research on the role of metacognitive processes taking place in creative thinking, research that can further elucidate the complexity of the creative process.

## Figures and Tables

**Table 1:** BEVoCI β coefficients representing cue validity in predicting originality scores and cue utilization for originality judgments in all conditions in Experiment 1 and Experiment 2.

	Originality Score	Originality Judgment	Originality Score	Originality Judgment
**Experiment 1a (Sidi et al. (2020), Experiment 2)**				
**Feedback manipulation:**	Rare		Common	
**Serial order**	0.28 ***	0.32 ***	0.28 ***	0.31***
**Number of words**	0.09 **	0.08 **	0.10**	0.13 ***
**Semantic distance**	0.41 ***	0.34 ***	0.38 ***	0.28 ***
**Response time**	0.06 *	0.09 **	0.06 *	0.11 ***
**Experiment 1b (Sidi et al. (2020), Experiment 3)**				
**Anchor manipulation:**	High		Low	
**Serial order**	0.28 ***	0.31 ***	0.46 ***	0.46 ***
**Number of words**	0.07 **	0.16 ***	0.06 *	0.08 **
**Semantic distance**	0.49 ***	0.29 ***	0.46 ***	0.34 ***
**Experiment 2—Semantic Distance Manipulation: Close**				
**Originality Manipulation:**	High		Low	
**Serial order**	0.23 ***	0.39 ***	0.31 ***	0.48 ***
**Number of words**	0.04 *	0.11 ***	0.07 ***	0.13 ***
**Semantic distance**	0.36 ***	0.18 ***	0.36 ***	0.20 ***
**Response time**	**−0.01**	0.03 **	0.03	0.07 ***
**Experiment 2—Semantic Distance Manipulation: Far**				
**Originality Manipulation:**	High		Low	
**Serial order**	0.27 ***	0.42 ***	0.29 ***	0.48 ***
**Number of words**	0.05 *	0.12 ***	0.09 ***	0.11 ***
**Semantic distance**	0.40 ***	0.16 ***	0.48 ***	0.16 ***
**Response time**	0.04 *	0.06 **	0.03	0.03

Note. Significance of a cue as a predictor (difference from zero), *** *p* ≤ 0.001; ** *p* ≤ 0.01; * *p* ≤ 0.05. **Bold** fonts: Significant mismatch between the association of the cue with originality scores and with originality judgments, *p* < 0.05.

**Table 2:** The examples used for instruction manipulations of originality (low, high) and distance (far, close).

Object	Hanger		Wine cork		Bucket	
Originality Semantic Distance	Low	High	Low	High	Low	High
Far	Grabber tool	Lighting fixture	Bottle drinks	Produce fire	Drinking water	Shower
Close	Hanging clothes	Plant support	Floating boat	Pin sticking	Wheelbarrow	Shovel

**Table 3:** Regression results of comparisons among the four groups in the β of the biasing factors (interaction effects between the group and the cue) that appear in Figure 2^1^.

Manipulation	Semantic Distance	Originality	Interaction
Dependent Variable (Figure 2 Panel)			
	**Serial Order**		
Originality Scores (Panel A)	*t*(9911) = 2.29, ***p* = 0.022**	*t*(9911) = 4.52, ***p* < 0.00**1	*t*(9911) = 2.11, ***p* = 0.035**
Originality Judgments (Panel B)	*t*(9911) = 1.55, *p* = 0.120	*t*(9911) = 5.94, ***p* < 0.001**	*t*(9911) = 2.33, ***p* = 0.020**
	**Semantic Distance**		
Originality Scores (Panel C)	*t*(9911) = 2.81, ***p* = 0.005**	*t*(9911) = 1.74, *p* = 0.08	*t*(9911) = 0.48, *p* = 0.630
Originality Judgments (Panel D)	*t*(9911) = 0.025, *p* = 0.799	*t*(9911) = 3.68, ***p* < 0.001**	*t*(9911) = 3.20, ***p* < 0.001**

Note. **Bold** fonts indicate a significant effect.

## Data Availability

Materials, data, BEVoCI paper, and analyses’ R code for Experiment 2, which includes newly collected data, are available in OSF: https://osf.io/t6wby/?view_only=16049b17a58b4cf48ab0ba39df48f483 (accessed on 9 January 2023).

## Note

1 These regressions were performed for each cue separately, without the other cues, for simplicity of the models.
